# Critical role of the SPAK protein kinase CCT domain in controlling blood pressure

**DOI:** 10.1093/hmg/ddv185

**Published:** 2015-05-20

**Authors:** Jinwei Zhang, Keith Siew, Thomas Macartney, Kevin M. O'Shaughnessy, Dario R. Alessi

**Affiliations:** 1 MRC Protein Phosphorylation and Ubiquitylation Unit, College of Life Sciences, University of Dundee, Dow Street, Dundee DD1 5EH, Scotland and; 2 Experimental Medicine and Immunotherapeutics, Department of Medicine, University of Cambridge, Cambridge, UK

## Abstract

The STE20/SPS1-related proline/alanine-rich kinase (SPAK) controls blood pressure (BP) by phosphorylating and stimulating the Na-Cl (NCC) and Na-K-2Cl (NKCC2) co-transporters, which regulate salt reabsorption in the kidney. SPAK possesses a conserved carboxy-terminal (CCT) domain, which recognises RFXV/I motifs present in its upstream activator [isoforms of the With-No-lysine (K) kinases (WNKs)] as well as its substrates (NCC and NKCC2). To define the physiological importance of the CCT domain, we generated knock-in mice in which the critical CCT domain Leu502 residue required for high affinity recognition of the RFXI/V motif was mutated to Alanine. The SPAK CCT domain defective knock-in animals are viable, and the Leu502Ala mutation abolished co-immunoprecipitation of SPAK with WNK1, NCC and NKCC2. The CCT domain defective animals displayed markedly reduced SPAK activity and phosphorylation of NCC and NKCC2 co-transporters at the residues phosphorylated by SPAK. This was also accompanied by a reduction in the expression of NCC and NKCC2 protein without changes in mRNA levels. The SPAK CCT domain knock-in mice showed typical features of Gitelman Syndrome with mild hypokalaemia, hypomagnesaemia, hypocalciuria and displayed salt wasting on switching to a low-Na diet. These observations establish that the CCT domain plays a crucial role in controlling SPAK activity and BP. Our results indicate that CCT domain inhibitors would be effective at reducing BP by lowering phosphorylation as well as expression of NCC and NKCC2.

## Introduction

SPAK (SPS1-related proline/alanine-rich kinase) and OSR1 (oxidative stress-responsive kinase 1) are closely related protein kinases, which play key roles in regulating cellular ion homeostasis and blood pressure (BP) (1,2). SPAK and OSR1 are activated following the phosphorylation of their T-loop residue (SPAK Thr233 and OSR1 Thr185) by one of the four isoforms of the WNK [with no lysine (K) kinase] protein kinase (3,4). The activity of SPAK and OSR1 is further enhanced following interaction with the scaffolding protein termed MO25 (5). The best-characterised SPAK/OSR1 substrates comprise the SLC12A (solute carrier family 12) family of electroneutral CCCs (cation–Cl co-transporters) (6–11). These transporters regulate intracellular chloride concentration critical in controlling BP and cell volume homoeostasis (12,13). SPAK/OSR1 protein kinases drive chloride influx by phosphorylation and activating sodium-driven CCC members. These include the NCC (Na–Cl co-transporter) in the distal convoluted tubule of the kidney (10), the NKCC2 (Na–K–2Cl co-transporter 2) in the thick ascending limb (TAL) of the kidney (9) and the ubiquitously expressed NKCC1 (6–8). SPAK/OSR1 also phosphorylate and inhibit potassium-driven CCCs that drive chloride efflux (11), which comprise four different K–Cl− co-transporters (KCC1–KCC4) (13,14). This reciprocal regulation of Na^+^- and K^+^-driven CCCs by SPAK and OSR1 ensures that cellular Cl^−^ influx and efflux is tightly co-ordinated (13,14).

The importance of the WNK signalling pathway is exemplified by its evolutionary conservation from worms to humans and that several Mendelian hypertension disorders in humans are caused by mutations in WNK pathway components (15,16). These include various mutations that lead to increased expression of the WNK1 and WNK4 genes causing PHAII [PseudoHypoAldosteronism type II, OMIM (17–23)]. Conversely, loss-of-function mutations in NCC and NKCC2 cause familial forms of hypotension and hypokalaemia termed Gitelman (OMIM #263800) and Bartter type 1 syndrome (OMIM #601678), respectively (24). A mutation that ablates the key activating WNK-regulated SPAK/OSR1 phosphorylation site on NCC [T60M (10)] also causes Gitelman's syndrome (25,26). Moreover, SPAK-knockout mice (27–29) or knock-in mice expressing a form of SPAK that cannot be activated by WNK kinase isoforms (30) exhibit low BP and are resistant to hypertension when crossed with animals bearing a PHAII-causing knock-in mutation that enhances WNK4 expression (31). Genome-wide association studies have also identified intronic SNPs within the SPAK gene (STK39) that correlate with increased BP in humans (32). Two commonly used drugs in medicine to lower high BP also target SPAK sodium-driven CCC substrates, namely thiazide diuretics (such as bendroflumethiazide) that inhibit NCC and the loop diuretics (such as furosemide) that inhibits NKCC2 (33,34).

These data suggest that chemical agents that inhibit SPAK would have the potential to treat hypertension, but without the off-target effects of agents like thiazide diuretics (16,35). One approach would be to elaborate small molecule compounds that directly inhibit SPAK/OSR1 protein kinase activity (36). However, to our knowledge, no highly selective and potent kinase inhibitors of SPAK and OSR1 have been reported. There is also concern whether sufficiently selective SPAK/OSR1 kinase inhibitors could be synthesized for the management of a chronic largely asymptomatic condition, without them inhibiting other protein kinases or ATP-binding enzymes and causing intolerable off-target effects. An alternative strategy to suppress SPAK/OSR1 function would be to target the docking domain within the non-catalytic C-terminal region of SPAK/OSR1 called the CCT (conserved C-Terminal) domain. *In vitro* and overexpression studies indicate that the CCT domain binds to conserved RFXV/I motifs present on WNK isoforms and that these interactions facilitate phosphorylation and activation of SPAK/OSR1 (37,38). Furthermore, the sodium-driven CCC members also possess conserved RFXI/V motifs at their N-terminus which experimental data indicate are critical for enabling SPAK/OSR1 to interact with, phosphorylate and stimulate activity of these transporters (9,10,38,39).

In this study, we sought to define the role that the CCT domain plays *in vivo* by generating and characterising SPAK knock-in mice in which function of the CCT domain has been ablated by a mutation of the critical Leu502 residue required for high-affinity binding of the RFXI/V motif. Our data demonstrate that the CCT domain of SPAK does indeed play an essential role in regulating the activation and function of SPAK. Our findings establish that SPAK CCT domain knock-in mice display markedly reduced phosphorylation and expression of NCC and NKCC2 in the kidney resulting in lower BP. Our data provide genetic validation that targeting the CCT domain would be therapeutically effective in reducing BP, by lowering phosphorylation as well as expression of NCC and NKCC2 in the kidney.

## Results

### Characterisation of a mutation that inhibits the CCT domain of SPAK

**Figure 1.**
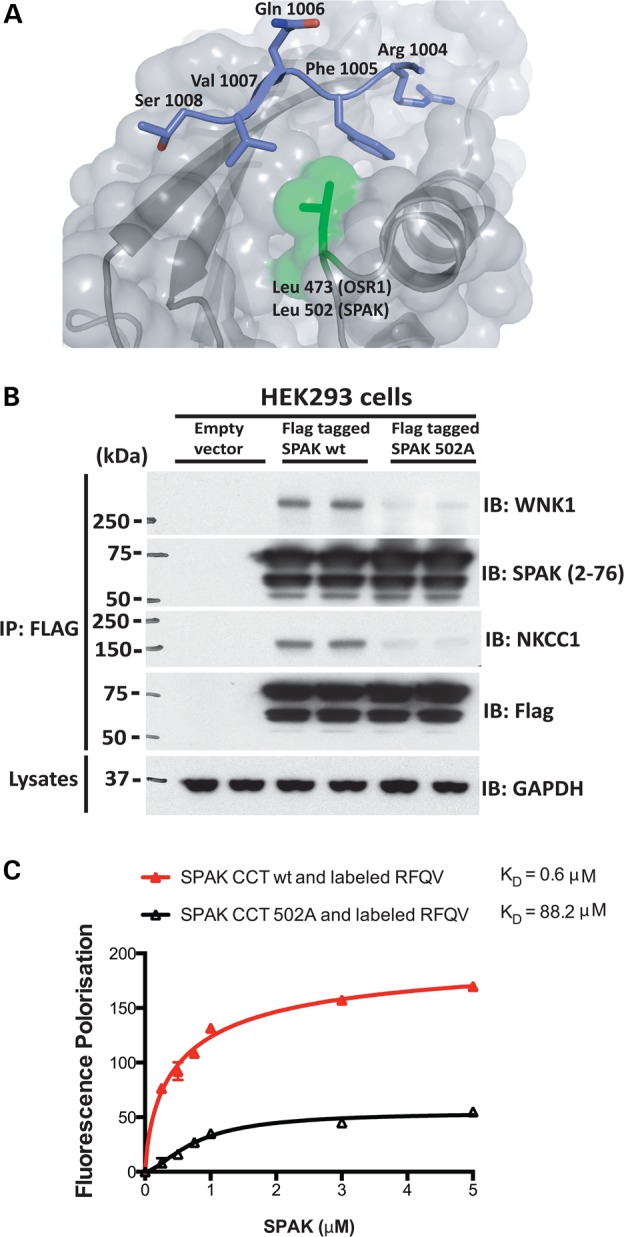
Evidence that SPAK associates with WNK1 and interaction is disrupted by SPAK CCT mutation. (**A**) Molecular interaction of the RFXV peptide with the conserved carboxy-terminal domain of OSR1 based on PDB 2V3S: CCT domain of OSR1 (residues 434–527) coloured in grey (β-strands and α-helices) bound to the GRFQVT WNK4-derived peptide coloured in blue. (**B**) HEK293 cells were transfected with constructs encoding a Flag empty vector or the indicated wild-type or mutant construct of N-terminal FLAG epitope-tagged full-length mouse SPAK. Thirty-six hours post-transfection, cells were lysed. Total cell extracts were subjected to immunoprecipitation (IP) with the indicated SPAK antibody, and immunoprecipitates were subjected to immunoblot (IB) analysis employing WNK1, SPAK and NKCC1 antibodies. Similar results were obtained in three separate experiments. (**C**) Analysis of SPAK–WNK interaction by fluorescence polarization. Purified human SPAK 452–547(end) and human SPAK 452–547 with L491A (equivalent to L502 in mouse) were diluted appropriately and mixed at a 1:1 volume ratio with 20 nm Lumino-Green-labelled WNK peptide to the concentration stated in the figure, with the peptide concentration consistent at 10 nm, and fluorescent polarization measurements were made. Binding curves, assuming one-site-specific binding, were then generated with Prism6 using milli-polarization (mP) units.

Previous crystallographic analysis of the human OSR1 CCT domain complexed to an RFXI motif-containing peptide derived from WNK4 (40) revealed that the highly conserved Leu473 CCT residue lying at the base of a deep hydrophobic pocket formed critical hydrophobic contacts with the Phe residue of the RFXI motif (Fig. 1A). Consistent with this, mutation of Leu473 to Ala reduced binding of OSR1 to RFXI WNK4 peptide over 100-fold (40). Mutation of the equivalent residue (Leu502) in mouse SPAK also prevented full-length mouse SPAK co-immunoprecipitating with endogenously expressed WNK1 and NKCC1 in HEK293 cells (Fig. 1B). Moreover, *in vitro* fluorescence polarisation studies confirmed that mutation of this Leu residue in the SPAK CCT domain reduced binding to an RFXV-motif-possessing peptide derived from WNK4 by ∼100-fold (Fig. 1C).

### Characterization of CCT domain-deficient SPAK^L502A/L502A^ mice

**Figure 2.**
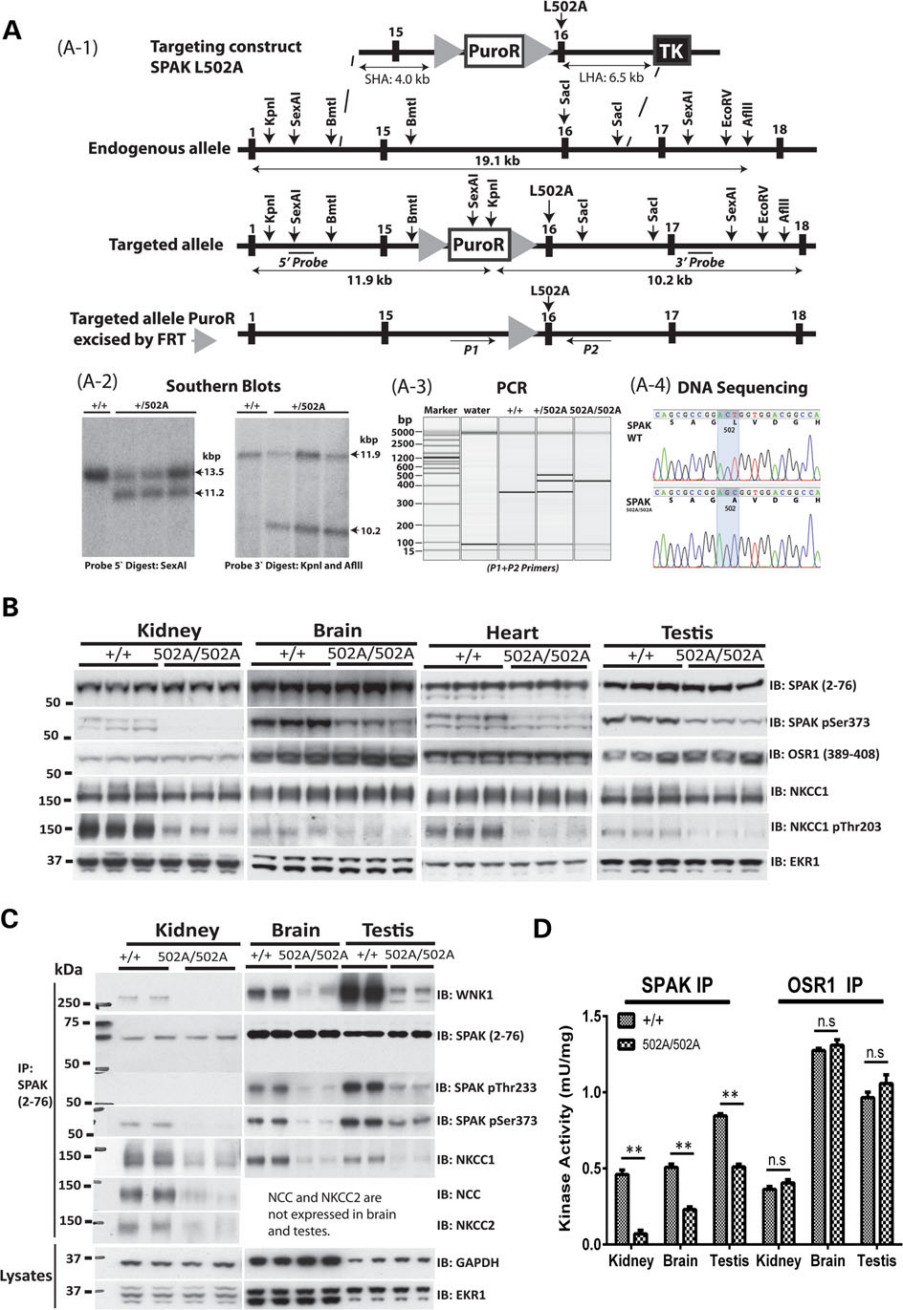
Targeting strategy used to generate SPAK knock-in mutations. (**A**-1) Diagram depicting the knock-in construct, the endogenous SPAK allele containing exons 15–16 and the targeted allele with the puromycin cassette removed by Flp recombinase. The black/grey rectangles represent exons, and the grey triangles represent FRT sites. Black lines with arrowheads are used to indicate the positions of the probes used for Southern analysis. The knock-in allele containing the Leu502Ala mutation in exon 16 is illustrated as a grey rectangle. The knock-in allele can be detected by genotyping using PCR primers P1 and P2, which are represented by short black lines with arrowheads. (**A**-2) Genomic DNA purified from the targeted ES cells from the indicated genotypes was digested with either the SexAI or Kpnl and Aflll and subjected to Southern analysis with the corresponding DNA probes. In the case of the 5′ probe, the wild-type allele generates a 13.5-kb fragment whereas the knock-in allele produces a 11.2-kb fragment. Similarly, the 3′ probe detects a fragment of 11.9 kb from the wild-type allele and 10.2 kb from the targeted knock-in allele. (**A**-3) Genomic DNA was PCR amplified with primers P1 and P2. The wild-type allele generates a 344-bp product whereas the knock-in allele generates a 419-bp product. The larger knock-in allele product is due to the presence of the 75-bp FRT site and flanking region, which remains in an intronic region following Flp-mediated excision of the puromycin resistance (PuroR) selection cassette, whereas the Thymidine kinase as a negative selection. (**A**-4) Genomic DNA purified from mice generated through heterozygous breeding was subjected to PCR to generate a product that encompasses the knock-in mutation region. The resultant PCR products were ligated into the pCR-Topo 2.1 vector and transformed into *E. coli*, and clones were sequenced. The wild-type and knock-in sequences are presented. (**B**) Expression of SPAK in tissues (kidney, brain, heart and testis) from wild-type and knock-in mice. The indicated tissue extracts (40 µg protein) from wild-type and SPAK502A/502A mice were subjected to immunoblot analysis with the specified antibodies. Immunoblots were run in parallel and exposed for the same amount of time to ensure that signal intensities can be directly compared. Similar results were obtained in three separate experiments. (**C** and **D**) Activity of SPAK in wild-type and knock-in mouse tissues. SPAK was immunoprecipitated from the kidney, testis and brain lysates from wild-type and SPAK^502A/502A^ mice using the SPAK-mouse peptide antibody. The immunoprecipitates were subjected to activity measurements using the CATCHtide peptide substrate (3). A fraction of the immunoprecipitates was also subjected to immunoblot assay with the indicated antibodies.

We exploited these observations to explore the impact that mutation of the CCT domain Leu502 residue had, by generating knock-in mice on an inbred C57BL/6J background, in which the Leu502 CCT domain residue was changed to Ala (Fig. 2A). Homozygous SPAK^L502A/L502A^ mice were born at the expected Mendelian frequency (Supplementary Material, Table S1), were of normal size and appearance and did not display any overt phenotype, at least up to 1 year of age (the oldest animals we have analysed).

Immunoblot analysis of tissues (kidney, brain, heart and testis) derived from littermate wild-type and SPAK^L502A/L502A^ homozygous animals of 2 months of age employing a novel mouse SPAK antibody that we generated that does not recognise mouse OSR1 (Supplementary Material, Fig. S1) revealed that the L502A mutation did not influence expression of SPAK in these tissues. We observed that all tissues analysed derived from SPAK^L502A/L502A^ mice displayed a significant reduction of the phosphorylation of SPAK at a key WNK phosphorylation site [Ser373 (3)], compared with wild-type mice (Fig. 2B). This is consistent with the L502A mutation suppressing the ability of WNK isoforms to phosphorylate SPAK. Furthermore, the SPAK^L502A/L502A^ knock-in animals displayed markedly reduced phosphorylation of NKCC1, in agreement with the CCT domain being required for SPAK to bind to and phosphorylate NKCC1 (Fig. 2B).

To obtain further evidence that the L502A mutation impacted on the ability of SPAK to bind WNK1 and NKCC1, we immunoprecipitated SPAK from kidney, brain and testis, derived from littermate wild-type and SPAK^L502A/L502A^ homozygous animals of 2 months of age and tested how CCT domain mutation effected in association with WNK1 and NKCC1. This revealed that in the SPAK^L502A/L502A^ knock-in animals, co-immunoprecipitation of WNK1 and NKCC1 with SPAK was markedly reduced in all tissues analysed (Fig. 2C). In the kidney, we also observed that mutation of the CCT domain inhibited co-immunoprecipitation of NCC and NKCC2 with SPAK (Fig. 2C). Further immunoblot analysis of SPAK immunoprecipitates confirmed that the L502A mutation inhibited phosphorylation of SPAK at the two key residues that WNK isoforms phosphorylate [Thr243 and Ser373 (3)] (Fig. 2C). SPAK as well as OSR1 immunoprecipitates from tissues (kidney, brain and testis) were also subjected to a protein kinase assay activity assay employing the previously described CATCHtide peptide that encompasses the NKCC1 phosphorylation sites (37). This revealed that SPAK immunoprecipitated from all tissues derived from SPAK^L502A/L502A^ knock-in mice displayed significantly reduced activity compared with wild-type animals (Fig. 2D). As expected, the activity of OSR1 was not affected in SPAK^L502A/L502A^ knock-in mice (Fig. 2D).

### SPAK^L502A/L502A^ mice display reduced phosphorylation of NCC and NKCC2 in kidney

**Figure 3.**
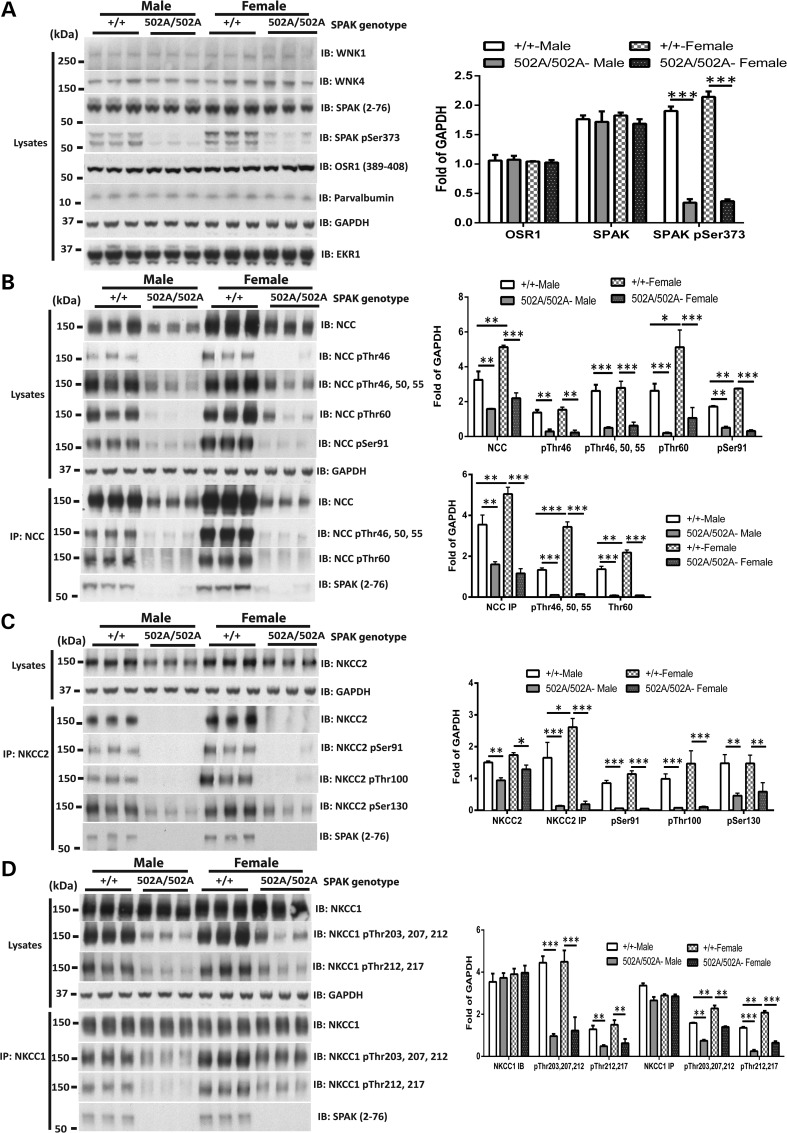
Reduced phosphorylation of SPAK, NCC, NKCC1, NKCC2 and expression of NCC and NKCC2 in both male and female SPAK^L502A/L502A^. (**A**) Expression of WNK1, WNK4, SPAK, OSR1 and Parvalbumin and phosphorylation of SPAK in wild-type and knock-in male and female mouse kidney. Kidney extract derived from the indicated mice was subjected to immunoblot analysis with the indicated antibodies. Each sample was derived from a separate littermate animal. Band intensities were quantified using Li-Cor Odyssey, and the results are presented relative to the expression of GAPDH. This study has been repeated over three times each with kidney extracts derived from different animals on each occasion with consistent results. (**B**) The right panel shows quantification of the results of the blots (*n* = 6, mean ± SEM). *P < 0.05; **P < 0.01; ***P < 0.001. (**C**) Analysis of kidney NCC protein levels and phosphorylation in male and female wild-type and homozygous knock-in mice following NCC immunoblot and immunoprecipitation. The upper panel shows that NCC from the indicated kidney extracts was subjected to immunoblot analysis with the total NCC antibody and a phosphospecific antibodies recognizing NCC phosphorylated at Thr46, 50, 55, 60 and Ser91 (Thr60 is a key SPAK/OSR1 phosphorylation site in NCC is frequently detected in Asian patients with Gitelman's syndrome). The down panel shows that NCC was immunoprecipitated from the indicated kidney extracts and subjected to immunoblot analysis with the total NCC antibody and a phosphospecific antibodies recognizing NCC phosphorylated at Thr46, 50, 55 and 60. Band intensities were quantified using Li-Cor Odyssey, and the results are presented relative to the expression of GAPDH. This study has been repeated over three times each with kidney extracts derived from different animals on each occasion with consistent results. (**D**) The right panel shows quantification of the results of the blots (*n* = 6, mean ± SEM). *P < 0.05; **P < 0.01; ***P < 0.001. (**E**) Analysis of kidney NKCC2 protein levels and phosphorylation in male and female wild-type and homozygous knock-in mice following NKCC2 immunoprecipitation. NKCC2 was immunoprecipitated from the indicated kidney extracts and subjected to immunoblot analysis with the total NKCC2 antibody and a phosphospecific antibodies recognizing NKCC2 phosphorylated at Ser91, Thr100 and Ser130, a major SPAK phosphorylation site. Each sample is derived from a separate littermate animal. Band intensities were quantified using Li-Cor Odyssey, and the results are presented relative to the expression of GAPDH. The study has been repeated over three times each with kidney extracts derived from different animals on each occasion with consistent results. (**F**) The right panel shows quantification of the results of the blots (*n* = 6, mean ± SEM). *P < 0.05; **P < 0.01; ***P < 0.001. (**H**) Analysis of kidney NKCC1 protein levels and phosphorylation in male and female wild-type and homozygous knock-in mice following NKCC1 immunoblot and immunoprecipitation. The upper panel shows that NKCC1 from the indicated kidney extracts was subjected to immunoblot analysis with the total NKCC1 antibody and a phosphospecific antibodies recognizing NKCC1 phosphorylated at Thr203, 207, 212 and 217. The down panel shows that NKCC1 was immunoprecipitated from the indicated kidney extracts and subjected to immunoblot analysis with the total NKCC1 antibody and a phosphospecific antibodies recognizing NKCC1 phosphorylated at Thr203, 207, 212 and 217. Band intensities were quantified using Li-Cor Odyssey, and the results are presented relative to the expression of GAPDH. This study has been repeated over three times each with kidney extracts derived from different animals on each occasion with consistent results. (**I**) The right panel shows quantification of the results of the blots (*n* = 6, mean ± SEM). *P < 0.05; **P < 0.01; ***P < 0.001.

We next compared the relative levels of phosphorylation of NCC in total kidney extracts derived from male and female 2-month-old wild-type and SPAK^L502A/L502A^ knock-in littermate animals (Fig. 3A–D). We monitored phosphorylation of NCC employing different previously characterised phosphospecific antibodies recognising major SPAK NCC phosphorylation sites (Thr46, Thr50, Thr55, Thr60 and Ser91) (10). This revealed that in both male and female mice, the phosphorylation of NCC at all residues analysed was drastically reduced in kidney derived from SPAK^L502A/L502A^ knock-in mice compared with wild type (Fig. 3B). Similar to what was previously observed in SPAK kinase inactive (SPAK^T243A/T243A^) knock-in mice (30) as well as SPAK knockout mice (27–29), we also observed ∼2-fold reduction in total levels of NCC protein in kidney extracts of SPAK^L502A/L502A^ knock-in mice compared with wild type (Fig. 3B). In contrast, kidney mRNA levels of NCC were similar in wild-type and of SPAK^L502A/L502A^ knock-in mice (Supplementary Material, Fig. S2).

We next monitored NKCC2 expression and similarly to NCC observed that levels were reduced ∼2-fold in the kidney of SPAK^L502A/L502A^ knock-in male and female mice compared with wild-type animals (Fig. 3C). We immunoprecipitated NKCC2 and analysed its phosphorylation at three residues that SPAK phosphorylates [Ser91, Thr100 and Ser130 (9)] and found that phosphorylation of each of these residues was substantially reduced in both male and female kidney derived from SPAK^L502A/L502A^ knock-in animals (Fig. 3C). Kidney mRNA levels of NKCC2 were similar in wild type and of SPAK^L502A/L502A^ knock-in mice (Supplementary Material, Fig S2).

We also analysed NKCC1 and found that the levels of this co-transporter were unaffected in kidney extracts derived from wild-type and SPAK^L502A/L502A^ knock-in male and female mice (Fig. 3D). We immunoprecipitated NKCC1 and analysed its phosphorylation at four sites that SPAK phosphorylates [Thr203, Thr207, Thr212 and Thr217 (3,5)] and found that phosphorylation of these sites was markedly diminished in both male and female kidney extracts derived from SPAK^L502A/L502A^ knock-in animals compared with wild type. Kidney mRNA levels of NKCC1 were similar in wild-type and of SPAK^L502A/L502A^ knock-in mice (Supplementary Material, Fig. S2).

### SPAK^L502A/L502A^ mice show a marked reduction in immunostaining for phosphorylated NCC and NKCC2 without remodelling of kidney tubules

**Figure 4.**
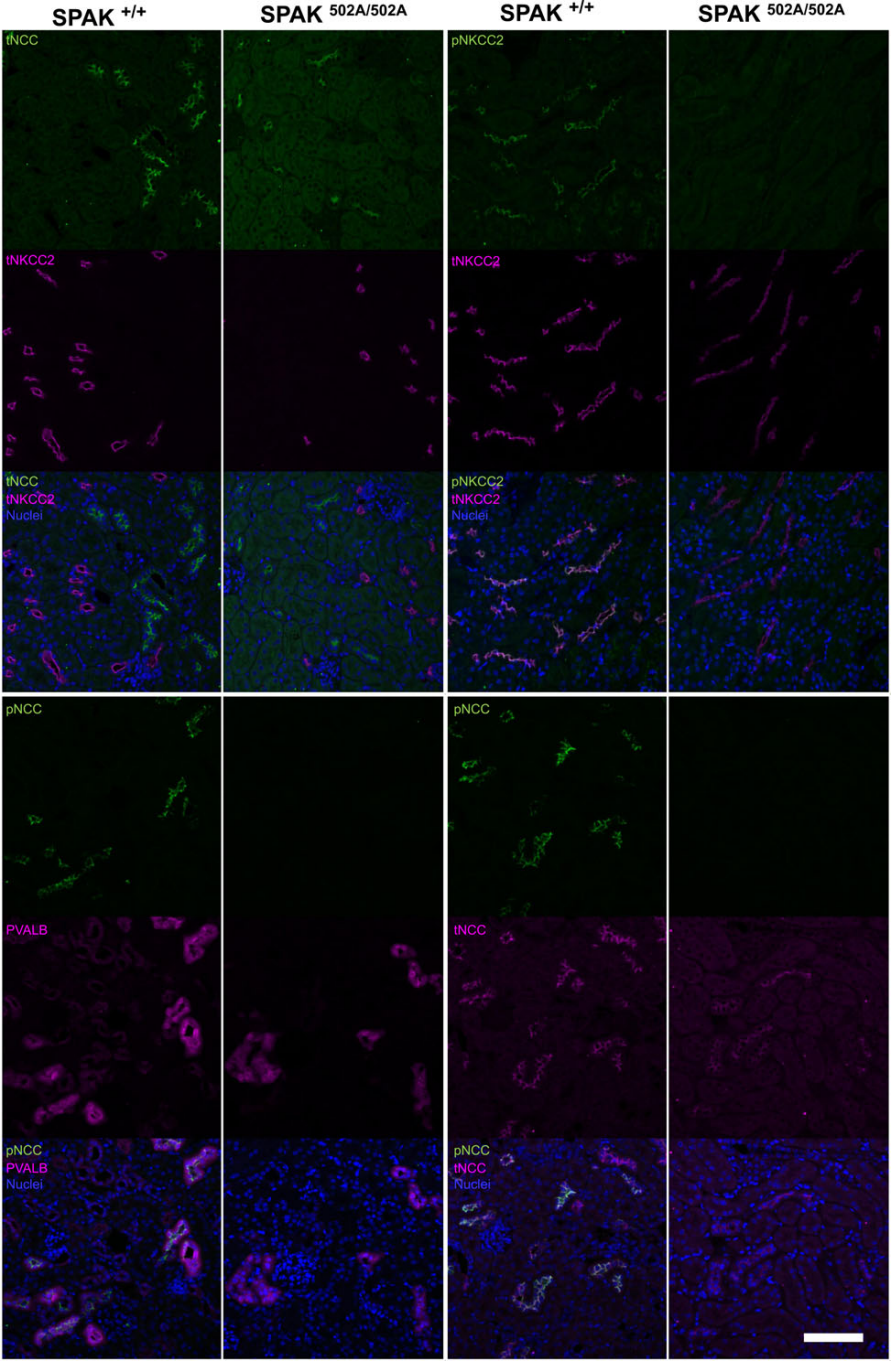
Immunolocalisation of NCC and NKCC2 in the renal tubules of SPAK^L502A/L502A^ Mice. Representative pseudocoloured average intensity z projections of immunofluorescent-stained kidney sections (*n* = 4 per genotype) showing the distribution of total and phospho proteins in the TAL marked by total NKCC2 (tNKCC2), early distal convoluted tubule (DCT1) marked by parvalbumin (PVALB) and whole distal convoluted tubule (DCT1/2) marked by total NCC (tNCC). Phospho-NCC T46 (pNCC) and phospho-NKCC2 S91 (pNKCC2) are virtually undetectable in the SPAK^L502A/L502A^ mice. Scale bar = 100 µm.

Confocal imaging of kidney sections from the SPAK^L502A/L502A^ mice confirmed the modest reduction in total NCC and NKCC2 in the tubules of the distal convoluted (DCT) and TAL tubules compared with wild-type mice, with no obvious changes in the morphology and numbers of tubules or the intracellular protein distribution between genotypes (Fig. 4). In striking contrast, staining for the phosphorylated forms of NCC and NKCC2 in the same tubules was almost completely lost in the SPAK^L502A/L502A^ mice in keeping with the immunoblots from whole kidney lysates (Fig. 3). Additionally no changes were observed in the intracellular localisation of WNK4 in the DCT or TAL (Supplementary Material, Fig. S3).

### SPAK^L502A/L502A^ mice display reduced BP and augmentation index

**Figure 5.**
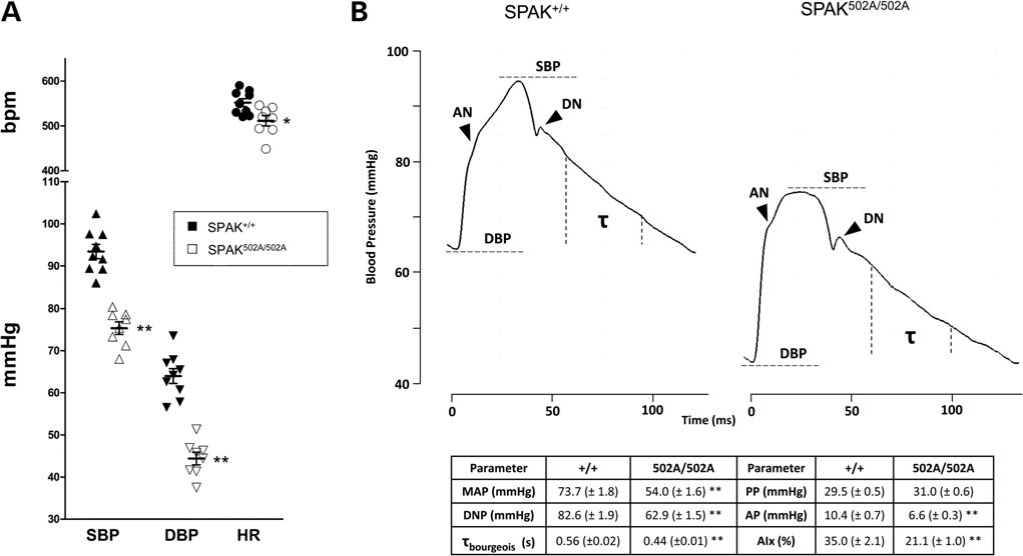
Blood pressure and pressure waveforms of SPAK^L502A/L502A^ mice. (**A**) Intravascular carotid measurements of systemic systolic blood pressure (SBP) and diastolic blood pressure (DBP), and heart rate (HR) determined by ECG R-R wave interval in mice under general anaesthesia. (**B**) Pulse waveform analysis reveals no change in pulse pressure (PP) (SBP–DBP) but does show decreased augmentation pressure (AP) (SBP–anacrotic notch [AN] pressure), dicrotic notch (DN) pressure and MAP (1/3 × SBP + 2/3 × DBP) in the SPAK^L502A/L502A^ versus wild type. This hypotensive phenotype is in part owing to changes in vascular contractility in SPAK^L502A/L502A^ mice as evidenced by their lower augmentation index (AIx) [AP/PP], a marker of arterial stiffness, and is further supported by a decrease in their diastolic pressure decay time constant (*τ*_bourgeois_) (1/slope of diastolic pressure decay; measured 30 ms after DN and 20 ms before end DBP), a surrogate marker of decreased vascular resistance. Bars are mean ± SEM, *n* = 8–9. Significant differences from the SPAK wild type are shown by: *P < 0.02, **P < 0.001.

To study the cardiovascular phenotype of these mice, we measured the arterial BP in the SPAK^L502A/L502A^ knock-in animals by carotid artery cannulation under general anaesthesia, which showed that their systolic, diastolic and mean arterial BPs were ∼20 mmHg lower than wild-type littermates (Fig. 5A). The lower BP of the SPAK^L502A/L502A^ knock-in animals was also reflected in a significantly lower left ventricular mass in these animals (Supplementary Material, Fig. S4).

Further analysis of the BP traces from the mice also showed that the shape of the arterial pressure wave was significantly different (Fig. 5B). The SPAK^L502A/L502A^ knock-in animals have a lower diastolic pressure time decay constant (*τ*_bourgeois_), an index of vascular resistance (41), suggesting decreased vascular tone compared with wild-type littermates (Fig. 5B). Specifically, the SPAK^L502A/L502A^ knock-in animals had lower augmentation of the peak systolic pressure (AIx) with no change in pulse pressure consistent with a reduction in arterial stiffness (42) (Fig. 5B).

### SPAK^L502A/L502A^ mice show a Gitelman syndrome pattern of plasma and urinary electrolytes and are salt wasting

**Figure 6.**
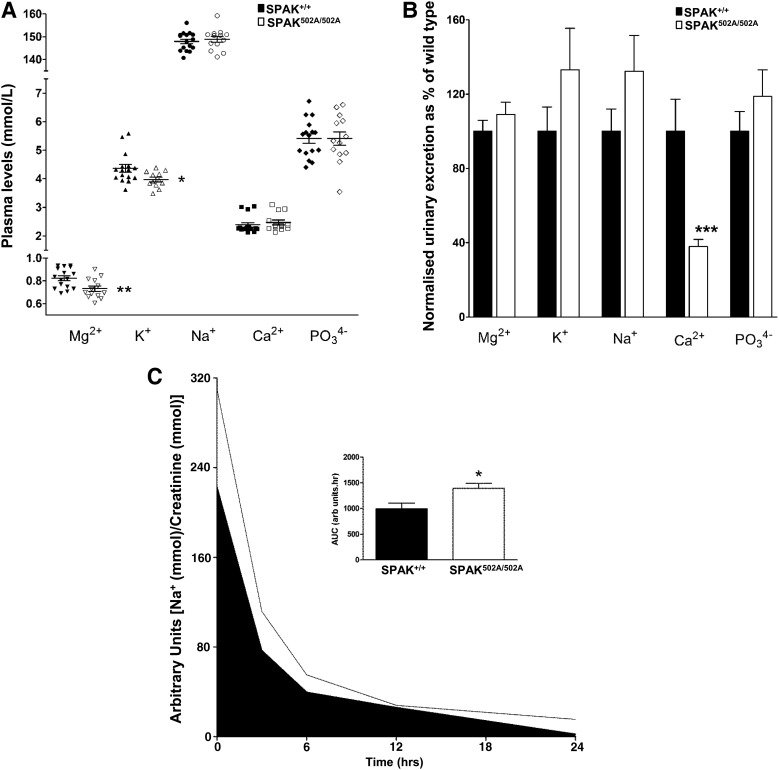
Plasma and urine electrolyte levels of SPAK^L502A/L502A^ mice. (**A**) Plasma electrolytes (*n* = 12–16 per genotype per electrolyte) and (**B**) creatinine normalised urinary electrolytes (*n* = 16–18 per genotype per electrolyte). (**C**) Urinary Na^+^ excretion expressed as mmol Na^+^/mmol creatinine at various time points after switching from a 3% w/w to a 0.03% w/w salt diet. Inset (C) shows the areas under the two curves (AUC) (*n* = 16 per genotype). Bars are mean ± SEM. Significant differences from the SPAK wild type are shown by: *P < 0.02, **P < 0.01, ***P < 0.005.

Analysis of the plasma electrolytes showed that the SPAK^L502A/L502A^ mice had a typical Gitelman pattern of plasma electrolytes with hypokalaemia and mild hypomagnesaemia compared with the wild-type animals (Fig. 6A). This is consistent with reduced WNK pathway signalling caused by the inactivation of the SPAK CCT domain. The urinary electrolytes also showed a Gitelman-like pattern of electrolytes with marked hypocalciuria compared with the wild-type mice (Fig. 6B). The salt wasting expected of a Gitelman-like phenotype was also confirmed by salt restricting the SPAK^L502A/L502A^ knock-in animals by switching to a 100-fold lower Na diet (Fig. 6C).

## Discussion

Our results define the importance that the CCT-docking domain plays in regulating the ability of WNK isoforms to control the activation as well as function of SPAK in regulating NCC/NKCC2 ion co-transporters and hence BP. Most importantly, our findings establish that a single-point mutation ablating the ability of the CCT domain to interact with RFXI motifs on its WNK activators or ion co-transporters substrates is sufficient to markedly reduce phosphorylation and levels of NCC/NKCC2 resulting in a ∼20 mmHg reduction in BP. Strikingly, the impact of the CCT domain mutation is similar to that observed previously by ablating SPAK kinase activity in knock-in mice (30) or by complete knockout of SPAK protein in mice (27–29). The SPAK^L502A/L502A^ animals still possess OSR1, which is activated normally by WNK isoforms (Fig. 2D), emphasising that inhibiting SPAK without effecting OSR1 is sufficient to markedly lower BP. This is consistent with previous analysis indicating that SPAK in mammals evolved as a result of a gene duplication of OSR1, to undertake more specialized roles such as control of NCC/NKCC2 in the kidney and regulation of BP (1).

Intriguingly, in the SPAK^L502A/L502A^ knock-in animals in addition to a reduced phosphorylation of NCC/NKCC2, we observed that the levels of NCC and NKCC2 protein were significantly reduced (Fig. 3), under conditions which mRNA levels were unaffected (Supplementary Material, Fig. S2). Similar results were also observed when analysing catalytically inactive SPAK knock-in mice (30). In converse, in mouse models in which the WNK signalling pathway is activated by introducing knock-in mutations that inhibit the ubiquitylation of and proteasomal degradation of WNK4 (i.e. WNK4 [D561A] knock-in mice (31,43) or KLHL3[R528H] knock-in mice (42)), NCC and NKCC2 protein levels are markedly elevated. Taken together results provide strong genetic evidence that phosphorylation of NCC/NKCC2 by SPAK in addition to regulating co-transporter activity also promotes the stability of these critical co-transporters. Further work is required to unravel the mechanism, by which NCC and NKCC2 expression is controlled by SPAK phosphorylation.

The plasma and urinary electrolyte pattern of the SPAK^L502A/L502A^ knock-in mice resembles Gitelman Syndrome. In fact, it recapitulates closely the phenotype of our previous report with a kinase-dead SPAK knock-in mouse (30). The extensive loss of NKCC2 and phospho-NKCC2 from the TAL tubules could be expected to produce a more extensive or even Bartter-like Syndrome (44). The levels of NKCC2 were also reduced in the kinase-dead SPAK knock-in mouse, but the reduction was modest in comparison with almost complete loss of phosphorylated NKCC2 from the SPAK^L502A/L502A^ mouse kidney (Figs 3 and 4). Nevertheless, the presence of both hypocalciuria and hypomagnesaemia in the SPAK^L502A/L502A^ mice is in keeping with a predominant loss of NCC function, because the loss of NKCC2 function in the mouse is associated with the opposite phenotype of hypermagnesaemia and hypercalciuria (45). In fact, plasma magnesium levels and urinary calcium excretion are clinical measures used routinely to distinguish between Gitelman and the related Bartter syndrome (46). Our finding that the total level and phosphorylation of NKCC2 is reduced in the kidneys of the SPAK^L502A/L502A^ mouse is also notable because previous SPAK knockout models reported hypophosphorylated NCC in the DCT, but markedly hyperphosphorylated NKCC2 in the TAL with increases in total NKCC2 levels (27) or without a change in total NKCC2 levels (28,29). The authors suggested that this was due to the removal of an inhibitory isoform of SPAK (SPAK2) and showed that the hypophosphorylation of NCC was accompanied by physical reduction in numbers of DCT1 tubular segments. Similarly, other studies with mouse models of NCC genetic ablation (47) and knockout of the DCT1-specific calcium-binding protein, parvalbumin (48), have also shown atrophy of the DCT. SPAK has been reported to be directly involved in the stabilisation of OSR1 at the apical membrane in the DCT and SPAK knockout results in a significant reduction of parvalbumin expression (29). However, unlike the knockout models used in these studies, the SPAK scaffolding functions remain intact in the point-mutant knock-in SPAK^L502A/L502A^ and SPAK^T243A/T243A^ mice. This may explain why we observed no changes in the levels or staining of parvalbumin (Figs 3A and 4). It is, therefore, possible to postulate that these remodelling phenomena may largely be due to loss of key components of regulatory pathways which maintain normal tubular structure, rather than a simply loss of NCC activity.

The altered shape of the arterial pressure waveform in our SPAK^L502A/L502A^ mice (Fig. 5B) is consistent with increased vascular compliance and reduced wave reflection in the periphery as compared with the wild-type animals. This may be explained in part by a diminished cardiac output arising from possible reductions in cardiac contractility owing to loss of NKCC1 activity in the heart (49) (Fig. 2B) and the lower heart rates of the SPAK^L502A/L502A^ mice (Fig. 5A), because augmentation is negatively correlated with heart rate in humans (50). It has also been previously shown that intracranial administration of bumetanide can be used to inhibit the over activity of NKCC1 in the hypothalamic paraventricular nucleus of spontaneously hypertensive rats. The inhibition of NKCC1 in these hypertensive animals acts to reduce sympathetic vasomotor tone and lower their heart rates (51). A similar phenomenon may be occurring in the SPAK^L502A/L502A^ mice owing to reductions in brain phospho-NKCC1 (Fig. 2), thus providing a potential neurological explanation for their lower heart rate and reduced vascular tone.

However, SPAK directly affects vascular smooth muscle contraction through WNK1 (52) and WNK3 (53) signalling and NKCC1 (27), the activity of which is necessary for maintaining vascular smooth muscle tone (54), so it is likely that reduced augmentation also reflects hypophosphorylation of NKCC1 in blood vessels in a parallel fashion to our findings in the kidney and other tissues (Figs 2 and 3). This is further supported by the shorter *τ*_bourgeois_ found in SPAK^L502A/L502A^ mice, which implies reduced vascular resistance consistent with a reduction in vascular contractility. This suggests that targeting the CCT domain of SPAK may have an additional effect on BP through a direct reduction in vascular tone. It also suggests a reduced arterial stiffness without a change in pulse pressure (Fig. 4), and both are importantly independent risk factors for cardiovascular mortality (42).

There is increasing interest in targeting the WNK-SPAK/OSR1-signalling pathway as a new therapeutic strategy to treat hypertension (16,35). Our finding that ablation of the CCT domain function is sufficient to reduce BP to the same extent as a kinase ablating knock-in mutation (30) suggests that the CCT domain of SPAK plays a similarly important role as the kinase domain in controlling BP. Thus, inhibitors that prevent the CCT domain-recognising RFXV motifs should be as effective as compounds that target the protein kinase domain of SPAK in lowering BP. Structural analysis reveals that CCT domain recognises RFXV/I motifs by adopting a unique fold not observed on other proteins (40). This may indicate that CCT domain inhibitors might be inherently more specific than ATP competitive kinase inhibitors that would have the potential to target other protein kinases and/or ATP binding enzymes. For the treatment of a chronic largely asymptomatic condition such as hypertension, it is particularly important that therapies are as specific as possible in order to reduce poorly tolerated off-target side effects.

Owing to the high degree of homology between the CCT domain of SPAK and OSR1, it may be impossible to develop drugs that selectively inhibit SPAK. However, even a drug that partially inhibited both SPAK and OSR1 isoforms might reduce phosphorylation and expression of NCC and NKCC1/2 enough to lower BP without significant adverse effects. Thus far one CCT domain inhibitor termed STOCK 1S-50699 has been reported that has an IC50 of ∼3 µm, that is capable of inhibiting WNK-SPAK phosphorylating NKCC1 and NCC in mammalian cell lines (11,39). STOCK 1S-50699 is highly hydrophobic, displays poor solubility and cannot be used in animal models, but the data obtained so far provide evidence that the development of CCT domain inhibitors is feasible.

In conclusion, our results establish that the SPAK CCT domain plays a major role in regulating BP. Our data suggest that the SPAK CCT domain regulates BP by promoting SPAK activation by WNK isoforms that in turn enhance both activity and expression of the renal sodium co-transporters NCC and NKCC2 with potential concomitant effects on NKCC1 in the vasculature. The hypotensive phenotype of the SPAK^L502A/L502A^ mice validate the CCT domain as a promising new target for future anti-hypertensive agents. The lack of an overt phenotype in these mice also indicates that CCT domain inhibitors may be well tolerated.

## Materials and Methods

### Materials

Lumio Green, Colloidal Blue staining kit and precast SDS polyacrylamide BisTris gels were from Invitrogen. GL Biochem synthesized all peptides to a purity of >95%; peptide sequences were determined by mass spectrometry. Tissue-culture reagents were from Life Technologies. P81 phosphocellulose paper was from Whatman, and [γ-32P]-ATP was from PerkinElmer.

### Plasmids

C-terminal human SPAK, full length, N-, C-terminal mouse SPAKs were amplified employing SuperScript III (Invitrogen) from placenta total RNA (Stratagene) using appropriate oligonucleotides. The RT–PCR products were ligated into pCR2.1-TOPO vector and sequenced. The sequence-verified SPAKs were sub-cloned into bacterial (pGEX-6P-1) and mammalian (pCMV5) expression vectors using BamHI and NotI. Required amino acid mutations were introduced into the pCR2.1-TOPO clone using site-directed mutagenesis by QuikChange method (Stratagene) but substituting the Taq enzyme with KOD Hot Start DNA polymerase (Novagen). All DNA plasmids used in this study are listed in Supplementary Material, Table S1. Oligonucleotides were obtained from Invitrogen Life Sciences. DNA sequencing was performed by The Sequencing Service, College of Life Sciences, University of Dundee (www.dnaseq.co.uk). All recombinant proteins, plasmids and antibodies generated for the present study are available on request and are described in additional detail on our website for reagents (https://mrcppureagents.dundee.ac.uk/).

### Expression and purification of proteins in *Escherichia coli*

All pGEX-6P-1 constructs encoding expression of recombinant with N-terminal Glutathione-S-Transferase (GST) tags were transformed into BL21 *E. coli* cells, and 1-l cultures were grown at 37°C in Luria Broth containing 100 µg/ml ampicillin until the absorbance at 600 nm was 0.8. Isopropyl ß-d-thiogalactopyranoside (30 µm) was then added, and the cells were cultured for a further 18 h at 26°C. Cells were isolated by centrifugation, re-suspended in 40 ml of ice-cold lysis buffer and lysed in one round of freeze/thawing, followed by sonication (Branson Digital Sonifier; ten 15-s pulses with a setting of 45% amplitude) to fragment DNA. Lysates were centrifuged at 4°C for 15 min at 26 00 *g*. The GST-SPAK recombinant proteins were affinity-purified on 0.5 ml glutathione–Sepharose and eluted in buffer A containing 0.27 m sucrose and 20 mm glutathione.

### Fluorescence polarization

Fluorescence polarization measurements were performed at 25°C with purified SPAK proteins in 50 mm Tris–HCl, pH 7.5, 150 mm NaCl and 2 mm DTT. The concentration of the SPAK proteins was determined by measuring their absorbance at 280 nm and calculated using the molar absorption coefficient determined by the ProtParam Online tool (55). All peptides [SEEGKPQLVGRFQVTSSK (EP4543) and SEEGKPQLVGAFQVTSSK (EP4544)] contained an N-terminal linker required for conjugating to the Lumio Green fluorophore (CCPGCCGGGG) and were initially resuspended in 50 mm ammonium bicarbonate, pH 8. Peptide labelling was achieved by incubating 10 nm of each peptide in a 0.5 ml reaction mixture of 20 μm Lumio Green in 25 mm Tris–HCl, pH 7.5, 200 mm NaCl and 5 mm 2-mercaptoethanol. Reactions were left to proceed in the dark for 2 h. The peptides were dialysed for 4 h into 25 mm Tris–HCl, pH 7.5, 200 mm NaCl and 5 mm 2-mercaptoethanol using a Micro DispoDIALYZER with a 100-Da molecular-mass cut-off (Harvard Apparatus) and then for another 12 h with changed buffer. For fluorescence polarization, mixtures were set up containing the indicated concentration of protein, 10 nm Lumio-Green-labelled peptide in a final volume of 30 μl. All individual bindings were performed in duplicate with at least 12 data points per curve. Fluorescence polarization measures were made using a BMG PheraStar plate reader, with an excitation wavelength of 485 nm and an emission wavelength of 538 nm, and measurements were corrected to the fluorescent probe alone. Data analysis and graphing were then performed in GraphPad Prism6; a one-site-specific binding model was assumed (*Y* = *B*_max_ * *X*/[*K*_d_ + *X*]) and the fitted dissociation constant computed. All experimental bindings were repeated at least twice and comparable results to those shown in the present study were obtained.

### Cell culture and transfections

HEK293 (human embryonic kidney 293) cells were cultured on 10-cm-diameter dishes in DMEM supplemented with 10% (v/v) foetal bovine serum, 2 mm l-glutamine, 100 U/ml penicillin and 0.1 mg/ml streptomycin. For transfection experiments, each dish of adherent HEK293 cells was transfected with 20 μl of 1 mg/ml polyethylenimine (Polysciences) and 5–10 μg of plasmid DNA as described previously (56). Thirty-six hours post-transfection, cells were lysed in 0.3 ml of ice-cold lysis buffer/dish, lysates were clarified by centrifugation at 4°C for 15 min at 26 000*g* and the supernatants were frozen in aliquots in liquid nitrogen and stored at −20°C. Protein concentrations were determined using the Bradford method.

### Buffers

Lysis buffer was 50 mm Tris–HCl, pH 7.5, 1 mm EGTA, 1 mm EDTA, 50 mm sodium fluoride, 5 mm sodium pyrophosphate, 1 mm sodium orthovanadate, 1% (w/v) NP-40 or 1% (w/v) Triton X-100, 0.27 m sucrose, 0.1% (v/v) 2-mercaptoethanol and protease inhibitors (one tablet per 50 ml). Buffer A was 50 mm Tris–HCl, pH 7.5, 0.1 mm EGTA and 0.1% (v/v) 2-mercaptoethanol. TBS-Tween buffer (TTBS) was Tris–HCl, pH 7.5, 0.15 m NaCl and 0.2% (v/v) Tween-20. SDS sample buffer was 1× NuPAGE LDS sample buffer (Invitrogen), containing 1% (v/v) 2-mercaptoethanol.

### Antibodies

The following antibodies were raised in sheep and affinity purified on the appropriate antigen by the Division of Signal Transduction Therapy Unit at the University of Dundee: WNK1-total antibody (residues 2360–2382 of human WNK1, S62B), WNK4-total antibody (residues 1221–1243 of human WNK4, S064B), WNK4 N-terminal antibody (residues 1–14 of mouse WNK4, S726D), SPAK-mouse antibody (2–76 of mouse SPAK, S668D), OSR1 mouse antibody (389–408 of mouse OSR1, SAHLPQPAGQMPTQPAQVSL, S149C), SPAK/OSR1 (T-loop) phospho-Thr233/Thr185 antibody (226–238 of human SPAK or residues 178–190 of human OSR1, TRNKVRKpTFVGTP, S204C), SPAK/OSR1 (S-motif) phospho-Ser373/Ser325 antibody (367–379 of human SPAK, RRVPGSpSGHLHKT, which is highly similar to residues 319–331 of human OSR1 in which the sequence is RRVPGSpSGRLHKT, S670B), SPAK phospho-Thr233 antibody (226–238 of human SPAK, TRNKVRKpTFVGTP, S668B), NKCC1 phospho-Thr203 + Thr207 + Thr212 (residues 198–217 of human NKCC1, HYYYDpTHTNpTYYLRpTFGHNT, S763B), NKCC1 phospho-Thr 212 + Thr 217 (residues 208–223 of human NKCC1, YYLRpTFGHNpTMDAVPR, S063D), NKCC1-total antibody (residues 1–260 of shark NKCC1, S841B), NCC phospho-Thr46 + Thr50 + Thr55 antibody (residues 41–60 of human NCC phosphorylated at Thr46 + Thr50 + Thr55, HPSHLpTHSSpTFCMRpTFGYNT, S908B), NCC phospho-Thr46 antibody (residues 40–54 of human NCC phosphorylated at Thr46, SHPSHLpTHSSTFCMRRR, S241C), NCC phospho-Thr60 antibody (residues 54–66 of human NCC phosphorylated at Thr60, RTFGYNpTIDVVPT, S995B), NCC phospho-Ser91 antibody (residues 85–97 of human NCC phosphorylated at Ser91, CTLADLHpSFLKQEGRR, S996B), NCC-total antibody (residues 906–925 of human NCC, CHTKRFEDMIAPFRLNDGFKD, S965B), NKCC2 phospho-Thr100 (residues 94–106 of human NKCC2, NTYYLQpTFGHNTM, S431C), NKCC2 phospho-Ser130 (residues 123–137 of human NKCC2, GPKVNRPpSLLEIHEQ, S888C), NKCC2 phospho-Ser91 (residues 86–97 of human NKCC2, RRFHAYDpSHTNTYYRR, S451C), NKCC2-total antibody (residues 1–174 of human NKCC2, S838B), GST-total antibody (raised against the glutathione S-transferase protein, S902A) and ERK1 total antibody (full-length human ERK1 protein, S221B). The anti-GAPDH antibody (ab8245), anti-NCC-total [SLC12A3] (AB95302) and the anti-parvalbumin antibody (ab11427) were purchased from Abcam. Anti-PVALB (Parvalbumin PV25) from Swant. Anti-NKCC2-total (LS-C313275) from LifeSpan BioSciences. The anti-ERK1/2 antibody (9102) was purchased from Cell Signalling Technology. The anti-FLAG antibody (F1804) was purchased from Sigma–Aldrich. Secondary antibodies coupled to horseradish peroxidase used for immunoblotting were obtained from Pierce. Fluorochrome-conjugated secondary antibodies for immunofluorescent confocal microscopy were obtained from Life Technologies and Abcam. Pre-immune IgG used in control immunoprecipitation experiments were affinity purified from pre-immune serum using protein G-Sepharose.

### Immunoprecipitation and assay of SPAK

One milligram of clarified cell lysate was incubated with 5 µg of the SPAK/OSR1 (total) antibody conjugated to 5 µl of protein G-Sepharose and incubated for 2 h at 4°C with gentle agitation. The immunoprecipitates were washed twice with 1 ml of lysis buffer containing 0.5 m NaCl and twice with 1 ml of buffer A. The SPAK/OSR1 immunoprecipitates were assayed with the CATCHtide peptide substrate (RRHYYYDTHTNTYYLRTFGHNTRR) that encompasses the SPAK/OSR1 phosphorylation sites on NKCC1 (3). Assays were set up in a total volume of 50 µl in buffer A containing 10 mm MgCl_2_, 0.1 mm [γ32P]ATP and 300 µm CATCHtide (37). After incubation for 30 min at 30°C, the reaction mixture was applied onto P81 phosphocellulose paper, the papers were washed in phosphoric acid and incorporation of 32P-radioactivity in CATCHtide was quantified by Cerenkov counting.

### Immunoblotting and total-antibody immunoprecipitation

Cell lysates (15 µg) in SDS sample buffer were subjected to electrophoresis on polyacrylamide gels and transferred to nitrocellulose membranes. The membranes were incubated for 30 min with TBS-T containing 5% (w/v) skim milk. The membranes were then immunoblotted in 5% (w/v) skim milk in TBS-T with the indicated primary antibodies overnight at 4°C. Sheep antibodies were used at a concentration of 1–2 µg/ml. The incubation with phospho-specific sheep antibodies was performed with the addition of 10 µg/ml of the dephospho-peptide antigen used to raise the antibody. The blots were then washed six times with TBS-T and incubated for 1 h at room temperature with secondary HRP-conjugated antibodies diluted 5000-fold in 5% (w/v) skim milk in TBS-T. After repeating the washing steps, the signal was detected with enhanced chemiluminescence reagent. Immunoblots were developed using a film automatic processor (SRX-101; Konica Minolta Medical), and films were scanned with a 600-dpi resolution on a scanner (PowerLook 1000; UMAX). Figures were generated using Photoshop/Illustrator (Adobe). For total-antibody immunoprecipitation NCC, NKCC1 and NKCC2 were immunoprecipitated from indicated kidney extracts. A 2 mg aliquot of the indicated clarified kidney extract were incubated with 15 μg of the indicated total NCC, NKCC1 and NKCC2 antibodies conjugated to 15 μl of protein-G–Sepharose. Incubation was for 2 h at 4°C with gentle agitation, and the immunoprecipitates were washed three times with 1 ml of lysis buffer containing 0.15 m NaCl and twice with 1 ml of buffer A. Bound proteins were eluted with 1× lithium dodecyl sulphate (LDS) sample buffer.

### Generation and genotyping of SPAK knock-in mice

The knock-in mice were generated by TaconicArtemis (http://www.taconic.com/wmspage.cfm?parm1=1453) as described in Figure 2A. The knock-in mice were generated and maintained on an inbred C57BL/6J background. Genotyping was performed by PCR using genomic DNA isolated from tails or embryonic membranes. For the SPAK mice, Primer 1 (5′ TCT GTA AGC TCA TTT ATG TAG TCA CC 3′) and Primer 2 (P2: 5′ CAA GTG AGT GAG TGA ATA CAG CC 3′) were used to detect the wild-type and knock-in alleles as described in Figure 2A. The PCR programme consisted of 2 min at 95°C, 30 s at 95°C, 1 min at 60°C and 1 min at 72°C: 35 cycles; 10 min at 72°C. DNA sequencing was performed by The Sequencing Service, College of Life Sciences, University of Dundee, UK (www.dnaseq.co.uk).

Mice were maintained under specific pathogen-free conditions, and all procedures were carried out in accordance with the regulations set by the Universities of Cambridge and Dundee, and the United Kingdom Home Office.

### Sample preparation and immunostaining for imaging

Harvested mouse tissues were immersion fixed in fresh 4% (w/v) formaldehyde-PBS pH 6.9 for 16 h at 37°C and washed three times in PBS and stored at 4°C until paraffin embedded. Five-micrometre sections were deparaffinised in Histoclear (National Diagnostics) and rehydrated in graded methanol steps. An antigen retrieval step was performed with R-Universal buffer in the 2100 antigen retriever for a single heat-pressure cycle (Aptum Biologics). Sections were permeabilised with 0.05% (v/v) Triton X-100–PBS for 20 min and blocked for 1 h at 37°C with 2% (v/v) donkey serum in 0.05% (v/v) Triton X-100–PBS. Primary antibodies were incubated overnight for 16 h at 4°C at the following concentrations diluted in 1% (v/v) donkey serum in 0.05% (v/v) Triton X-100–PBS: 2 μg/ml for pNCC T46, tNCC, pNKCC2 S91, tNKCC2 and 4 μg/ml for WNK4 N-terminal and 1:2000 for PVALB. Phospho-specific antibodies included the addition of 10 μg/ml of the non-phospho peptide used to raise the antibody per 2 μg/ml of antibody used. Negative controls omitted the primary antibody and were processed in parallel (Supplementary Material, Fig. S3). Slides were then washed for 20 min in 0.05% (v/v) Triton X-100–PBS and incubated in secondary antibody for 1 h at 37°C. Pre-absorbed donkey IgG-conjugated Alexa Fluor 488, 633 and 647 secondary antibodies (Life Technologies/Abcam) were used at 1:200 diluted in 1% (v/v) donkey serum in 0.05% (v/v) Triton X-100–PBS for immunofluorescent labelling. Slides were washed as mentioned earlier, counterstained using Sytox orange nucleic acid stain (S11368—Life Technologies) and mounted using Prolong gold antifade (P36930—Life Technologies) and shielded from light.

### Image acquisition and processing

Immunofluorescent images were acquired on the Leica TCS SP2 laser-scanning confocal with 488-, 543-, 633-nm laser lines mounted on an upright Leica DM RXA fluorescent microscope using an HC PL FLUOTAR 20X/0.5NA objective. Acquisition parameters were as follows: 12-bit, 1024 × 1024 pixels, 2× digital zoom, 800 Hz scan speed, 4-line Kalman filtering, sequential (by line) channel imaging and 10 slice z-stack of 5 µm.

In FIJI image analysis software, fluorescent z-stacks underwent background subtraction (1000px radius rolling ball, no smoothing) and average intensity z-projection. Brightness and contrast were adjusted by linear histogram stretching to enhance visibility. Any images to be compared with one another were processed evenly across the whole image in parallel, the exception to this were nuclei images where brightness and contrast were performed on each image independently to ensure the best visibility.

### *In vivo* pulse waveform analysis and BP measurements

Animals were anaesthetized with isoflurane on 100% O_2_ (induction: 3%, maintenance: 1.75%) and placed on a self-regulating rectal probe-coupled heat mat (TC-1000; CWE) to maintain a body temperature of 37°C. Heart rate was measured by the R-R wave interval from ECG Lead II using an animal bio amp (FE136; AD Instruments) with needle electrodes inserted into fore and hind limbs (MLA1213; AD Instruments). The right carotid artery was catheterized with a 1F Mikro-Tip pressure transducer (SPR-1000; Millar) connected to a bridge amp (FE221; AD Instruments) and powerlab system (PL3504/P; AD Instruments). When the animals had stabilized, measurements were taken at 2000 samples/s using lab chart version 7/8 pro (AD Instruments) to record ECG and BP pulse waveforms.

Data processing and analysis was performed in Lab chart 8 pro. Using the BP add-on, systolic, diastolic and dicrotic notch BPs were automatically detected per beat (beats with respiratory-induced artefact were gated out of the analysis using the beats classifier). Mean arterial pressure (MAP) was calculated as (1/3 systolic pressure + 2/3 diastolic pressure) and pulse pressure as (systolic pressure − diastolic pressure). A macro was scripted to detect the anacrotic notch, by using the third zero value crossing the fourth derivative of the pressure (57). The augmentation pressure was calculated as (systolic pressure − anacrotic notch pressure) and augmentation index as (augmentation pressure/pulse pressure). A macro was scripted to measure the slope of the diastolic pressure decay, 30 ms after the dicrotic notch and 20 ms before the end diastolic pressure (to avoid perturbations caused by aortic valve opening/closing). The reciprocal of this slope (ignoring the sign) was calculated from the 20% trimmed mean values to determine the time decay constant of the diastolic pressure decay (*τ*_bourgeois_), which correlates with vascular resistance (41). Data collection and analysis was carried out in a blinded fashion throughout.

### Plasma and urine electrolyte measurements

Animal were placed on a 3% w/w Na diet for 14 days with urine and plasma samples collected on between Days 7 and 10 for baseline. On Day 14, mice were switched onto a 0.03% w/w Na diet with urine samples collected at 0, 3, 6, 12, 24 h after Na-diet switch. Spot urine was collected from awake mice following spontaneous micturition on handling over a sheet of Saran^®^ wrap or Parafilm^®^. Samples were then divided and one sample received acidification with HNO_3_ to a final concentration of 1% v/v to prevent precipitation of electrolytes, before both were stored at −80°C. Blood was collected by saphenous venepuncture in awake restrained animals, and plasma was separated by using Microvette^®^ CB 300 LH (Sarstedt) centrifuged at 2000*g* for 5 min before storage at −80°C.

Plasma and urine (non-acidified) creatinine levels were assayed in the core Core Biochemical Assay Laboratory, Addenbrooke's Hospital, Cambridge, UK. Plasma and urine (acidified) samples were diluted 1:1000 using ultra-pure polished water containing 1% v/v HNO_3_. Cations were then measured using an inductively coupled plasma—optical emission spectrometry (Perkin Elmer ICP-OES Analyser) with known standards and pre-set elemental spectra. Data collection and analysis was carried out in a blinded fashion throughout.

### Statistical analysis

Data are presented as mean ± SEM, with n representing the number of analysed mice. Mean values were compared by the Student's *t*-test for paired or unpaired observations where appropriate, using SigmaStat Program (Jandel Scientific, Chicago, IL, USA) or GraphPad Prism5, or by ANOVA with *post hoc* testing using version 15 of SPSS software. A *P* < 0.05 was considered statistically significant.

## Supplementary Material

Supplementary Material is available at *HMG* online.

Supplementary Data
