# Correction to: Critical role of the SPAK protein kinase CCT domain in controlling blood pressure

**DOI:** 10.1093/hmg/ddaf133

**Published:** 2025-08-20

This is a correction to: Jinwei Zhang, Keith Siew, Thomas Macartney, Kevin M. O'Shaughnessy, and Dario R. Alessi, Critical role of the SPAK protein kinase CCT domain in controlling blood pressure, *Human Molecular Genetics*, Volume 24, Issue 16, 15 August 2015, Pages 4545–4558, https://doi.org/10.1093/hmg/ddv185.

In April 2025, the authors contacted the journal to request publication of corrected Figures 2 and 3. The authors were made aware of similarities between the Western blot bands for NKCC1 (Kidney) and NKCC1 (Testis) in Figure 2B. They were also made aware that there were similarities in bands in Figure 3. The bands for NKCC2 pSer130 in Figure 3C are similar to the NKCC1 pThr221,227 bands in Figure 3D. Also, the bands for NCC pThr46 in Figure 3B are similar to the bands for NKCC2 pSer91 in Figure 3C. These concerns were also raised on PubPeer (see https://pubpeer.com/publications/C146E3726D22CA2C43023267F5F465).

After reviewing the raw data, the authors determined that during the final figure assembly, inadvertent errors in image selection occurred. The authors apologize for the errors and have provided corrected Figures 2B, 3C, and 3D which are published in this notice.

The Editors have carefully reviewed the raw data provided by the authors, approved the corrected Figures 2 and 3, and are satisfied that the changes do not affect the conclusions of the article.

These details have been corrected only in this correction notice to preserve the published version of record.


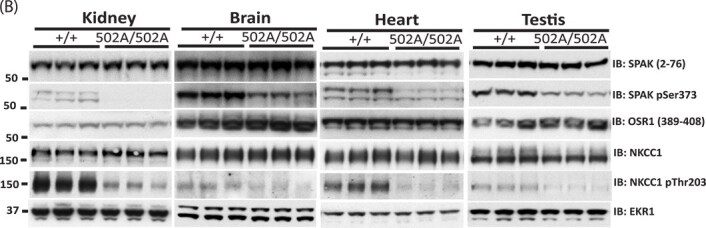


**Figure 2.**


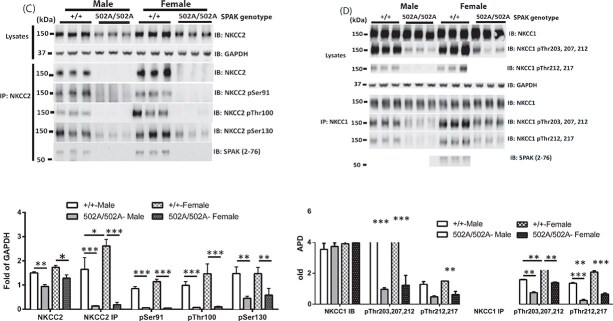


**Figure 3.**

