# Supplementary material for: Critical role of the SPAK protein kinase CCT domain in controlling blood pressure
Source: Hum Mol Genet. 2015 May 20;24(16):4545–58. doi: 10.1093/hmg/ddv185 (PMC4512625; doi:10.1093/hmg/ddv185)
Supplement: Supplementary Data [file supp_24_16_4545__index.html]

Critical role of the SPAK protein kinase CCT domain in controlling blood pressure — Critical role of the SPAK protein kinase CCT domain in controlling blood pressure — Critical role of the SPAK protein kinase CCT domain in controlling blood pressure — Supplementary Data 

# Critical role of the SPAK protein kinase CCT domain in controlling blood pressure

## Supplementary Data

Supplementary Data

- Supplementary Data - Pdf file
