# Supplementary material for: Critical role of the SPAK protein kinase CCT domain in controlling blood pressure
Source: Hum Mol Genet. 2015 May 20;24(16):4545–58. doi: 10.1093/hmg/ddv185 (PMC4512625; doi:10.1093/hmg/ddv185)
Supplement: Supplementary Data [file supp_ddv185_ddv185supp.pdf]

Supplementary Figure Legends

Figure S1. **Generation and characterisation of specific SPAK antibodies**. The N-SPAK antibody was generated by ligating cDNA corresponding to amino acids 2–74 of mouse SPAK downstream of the GST open reading frame in the pGEX-6P-1 vector. The resulting fusion protein was injected into sheep to produce polyclonal antibodies. HEK293 cells were transfected with the constructs encoding the full-length mouse FLAG-tagged SPAK wild type and SPAK L502A. 36 h post transfection, the lysates were subjected to immunoblotting with the indicated antibodies. The indicated amounts of recombinant mouse full-length GST-SPAK protein were subjected to immunoblot analysis on HEK293 cell lysates (A) and kidney lysates (B) with the newly generated SPAK and OSR1 mouse antibodies described in the Materials and Methods.

Figure S2. **Quantitative real-time PCR analysis of sodium ion co-transporter expression in the kidney**. Total RNA was purified from wild type and $SPAK^{L502A/L502A}$ mice kidneys using RNeasy kits (Qiagen) and reverse transcribed using iScript (Biorad). mRNA levels of SPAK, OSR1, NKCC1, NKCC2 and NCC were assessed by using Sybrgreen-based quantitative real-time PCR according to manufacturer's instructions. The levels were quantified from five separate mice and each PCR was performed in triplicate. Results were normalized to 18S rRNA expression and levels are presented relative to wild type expression. Primer sequences used are shown in Supplementary Table S3.

Figure S3. **Cardiac ventricular mass**. The weight of the Left Ventricle and Septum combined (LV+S) are expressed as a % of bodyweight (BW). Bars are mean±SEM n=14-16. Significant differences from the SPAK wild type are shown by: * $P<0.02$.

Figure S4. **Representative pseudocoloured average intensity z projections of immunofluorescent stained kidney sections**. (A) Immunolocalisation of WNK4 in the DCT marked by tNCC and TAL marked by NKCC2 (n=4 per genotype. Scale bar = 50μm). (B) Primary antibody negative controls for each staining set (Scale bar = 100μm).

Supplementary Table S1. **Breeding of SPAK knock-in mice**. Matings for SPAK were set up as outlined above and the progeny were genotyped as described in the methods. The percentage of each genotype observed is indicated in parenthesis followed by its expected Mendelian frequency.

Supplementary Table S2. **Summary of the purified proteins used in this study**. The DSTT DU number, purification system and affinity tags are used in Figure 1 and Figure S1.

Supplementary Table S3. **Primer sequences used for quantitative PCR undertaken in this study**.

Supplementary Figure S1

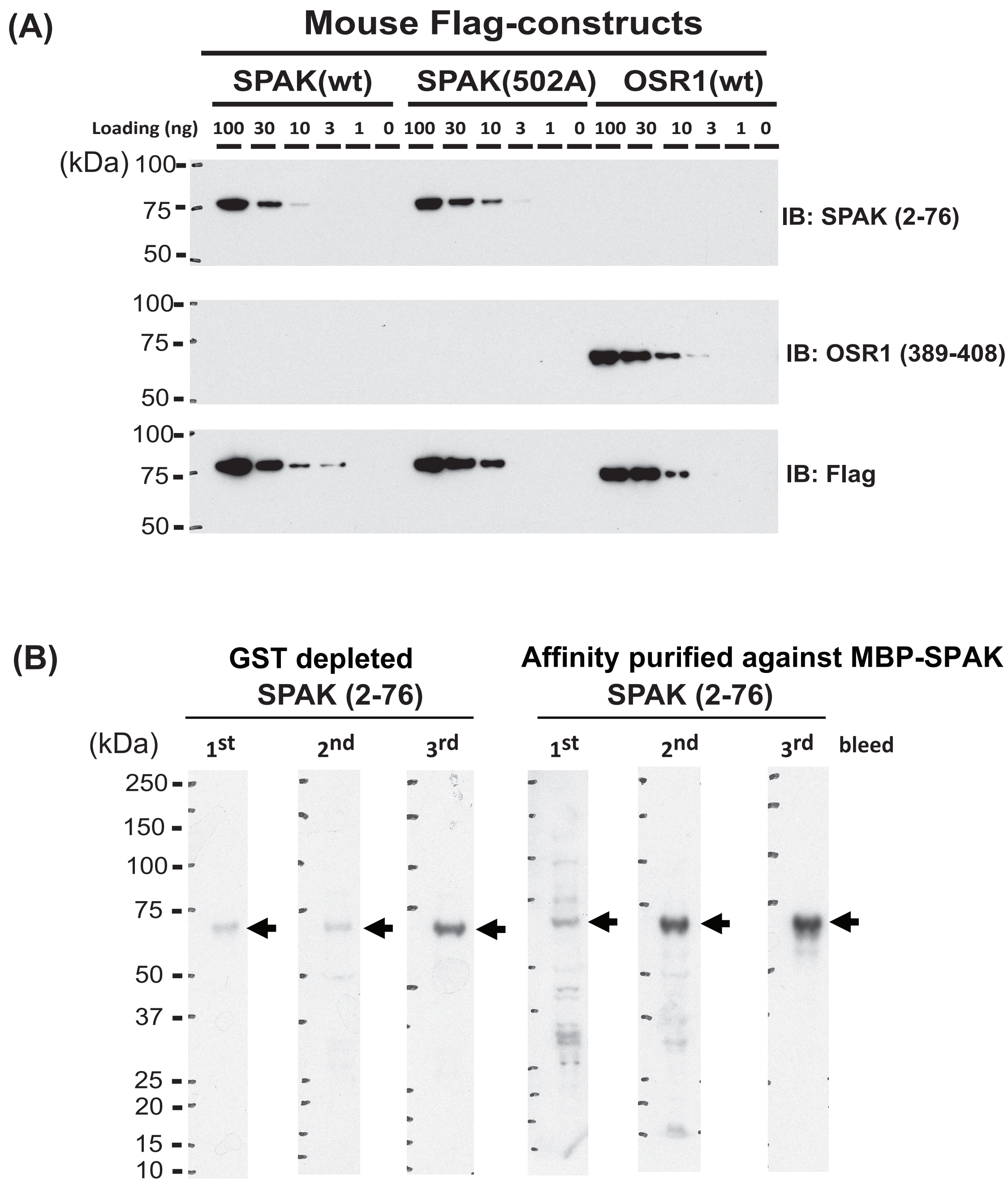

Supplementary Figure S2

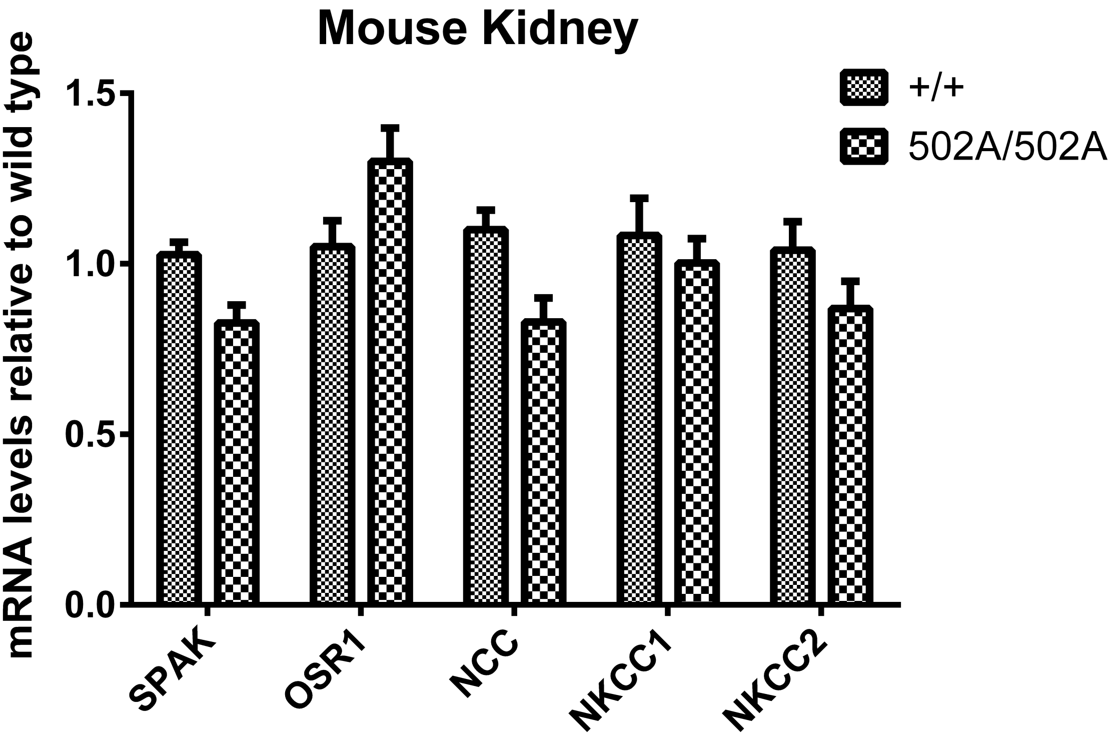

Supplementary Figure S3

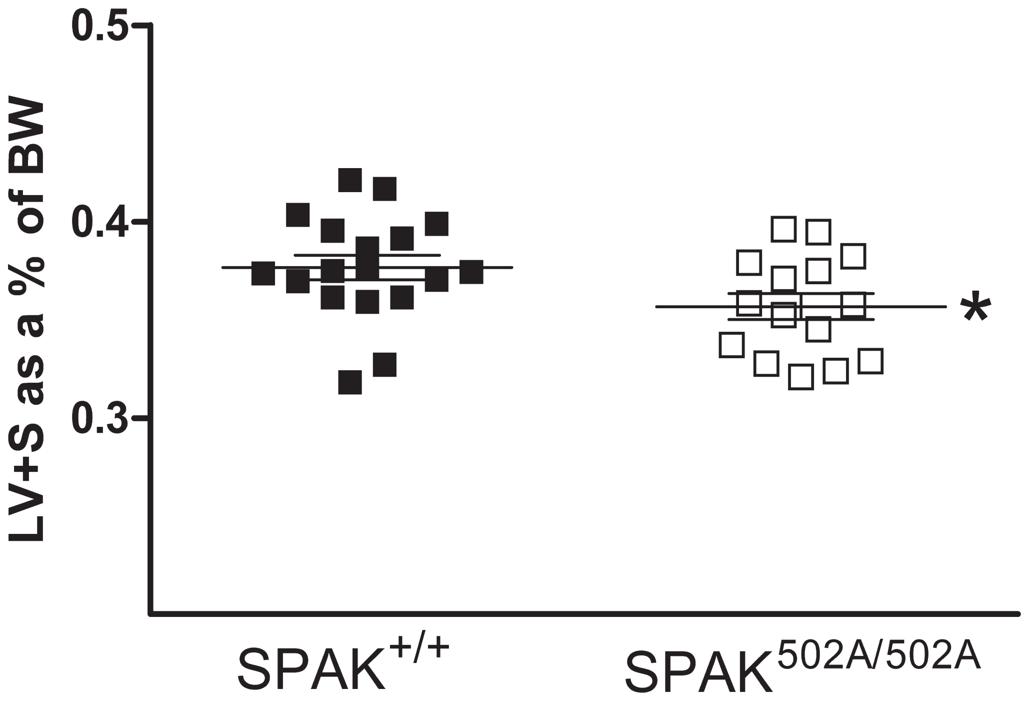

Supplementary Figure S4

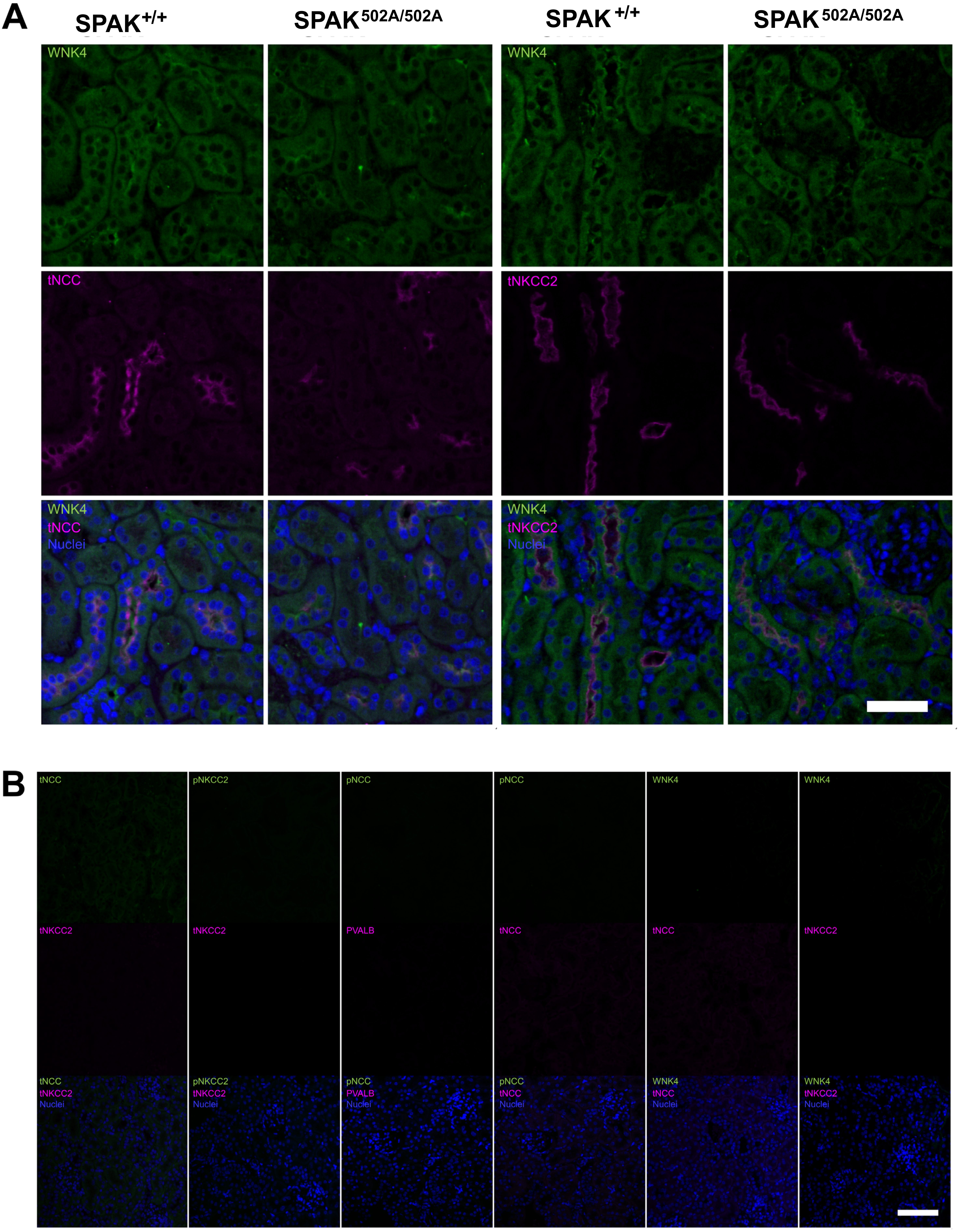

Supplementary Table S1

Cross	Genotype		Number Born (%)	Expected Mendelian Frequency (%)
SPAK (+/502A) OSR1 (+/+)	SPAK (+/+)	OSR1 (+/+)	94 (30%)	25
SPAK (+/502A) OSR1 (+/+)	SPAK (+/502A)	OSR1 (+/+)	180 (57%)	50
	SPAK (502A/502A)	OSR1 (+/+)	42 (13%)	25

Supplementary Table S2

Kinase/protein	DU number	Expression system	Affinity tag
GST-SPAK 452 – 545 (end)	DU41572	Bacteria	GST
GST-SPAK 452-end L491A	DU41619	Bacteria	GST
GST-mSPAK 2-74	DU44920	Bacteria	GST
GST-mSPAK 424-556	DU44891	Bacteria	GST
MBP-mSPAK 2-74	DU48033	Bacteria	MBP
MBP-mSPAK 424-556	DU48034	Bacteria	MBP
FLAG-mSPAK	DU48072	HEK293	FLAG
FLAG-mSPAK Opt L502A	DU48455	HEK293	FLAG

Supplementary Table S3

Symbol	Protein	Use	Sense primer (5`-3`)	Antisense primer (5`-3`)
SPAK	SPAK	PCR	TCTGTAAGCTCATTTATGTAGTCACC<br>P1: STK39 6062_39	CAAGTGAGTGAGTGAATACAGCC<br>P1: STK39 6062_40
SPAK	SPAK	q-PCR	GTCTAGGACATGATGGTATGG	CCAACATGGGGTACCAACAAATGC
OSR1	OSR1	q-PCR	CGCTGCAGTCTCCTGTCATCTG	CAGAGTGAGGTCTAGGACAGCCAGG
SLC12A1	NKCC2	q-PCR	TTGGATATAACCCACGCCTTTACG	GCCATGCCGCTGTTCATCTC
SLC12A2	NKCC1	q-PCR	GCAGATCCTCAGTCAGCCATAC	TCCCGAACAACACACGAACC
SLC12A3	NCC	q-PCR	CGGGGTTTGTGTCATGAGGATG	TTGTATCAATGCCTCTGGGGTTG
18S	18S rRNA	q-PCR	GTAACCCGTTGAACCCCATT	CCATCCAATCGGTAGTAGCG
